# Racial Disparities in Outcomes of Adults Hospitalized for Viral Pneumonia

**DOI:** 10.7759/cureus.11909

**Published:** 2020-12-04

**Authors:** Pius E Ojemolon, Valeria P Trelles-Garcia, Daniela Trelles-Garcia, Asim Kichloo, Sairam Raghavan, Abdulrahman I Abusalim, Precious Eseaton

**Affiliations:** 1 Anatomical Sciences, St. George's University, St. George's, GRD; 2 Internal Medicine, John H. Stroger, Jr. Hospital of Cook County, Chicago, USA; 3 Internal Medicine, Saint Francis Hospital, Evanston, USA; 4 Internal Medicine, Central Michigan University, Saginaw, USA; 5 College of Medicine, University of Benin, Benin City, NGA

**Keywords:** mortality, viral pneumonia, pulmonary disease, racial disparity, inpatient outcomes

## Abstract

Background

Viral pneumonia is an important cause of respiratory morbidity and mortality. Cases of viral pneumonia are becoming increasingly more common as at-risk populations increase globally. We sought to highlight the racial distribution of hospitalized patients with viral pneumonia and compare their outcomes.

Materials and methods

Data were obtained from the Nationwide Inpatient Sample (NIS) for 2016 and 2017. The study involved adults who had a principal discharge diagnosis of viral pneumonia. The primary outcome analyzed was inpatient mortality. Secondary outcomes included the development of sepsis, septic shock, acute respiratory failure, acute respiratory distress syndrome, non-ST segment elevation myocardial infarction (NSTEMI), acute kidney failure, deep vein thrombosis, pulmonary embolism, cerebrovascular accident, need for mechanical ventilation, and use of vasopressors as well as mean length of hospitalization and mean total hospital charges.

Results

Blacks and Hispanics had lower inpatient mortality adjusted odds (aOR: 0.39, 95% CI = 0.229 - 0.662, p<0.001 and aOR: 0.55, 95% CI = 0.347 - 0.858, p=0.009, respectively) compared to Whites. Black and Hispanic patients were also found to have lower adjusted odds ratio of having acute respiratory failure (aOR: 0.54, 95% CI = 0.471 - 0.614, p<0.001, and 0.66, 95% CI = 0.576 - 0.753, p<0.001, respectively).

Conclusion

Black and Hispanic patients are at lower risk of adverse outcomes when compared to White patients with viral pneumonia.

## Introduction

Community-acquired pneumonia (CAP) is one of the leading causes of morbidity and mortality worldwide. According to World Health Organization (WHO) estimates, 450 million cases of pneumonia are recorded globally, with approximately four million mortalities per annum. CAP is known to be particularly prevalent among children and the elderly [1]. In the United States, there were 1.3 million visits to emergency departments with pneumonia as the primary diagnosis in 2017, and the annual health care cost spent on CAP exceeds $10 billion [2,3]. Of the cases of pathogen-diagnosed CAP, viruses are identified in 13-50% of cases as sole pathogens while 8-27% of cases are mixed bacteria-virus infections [4,5].

The most common causes of viral pneumonia are influenza, respiratory syncytial virus (RSV), rhinoviruses, and coronaviruses. Less commonly implicated viruses include adenoviruses, parainfluenza virus, and the human metapneumovirus. Enzyme-linked immunosorbent assays (ELISA), immunofluorescence, and polymerase chain reaction (PCR) tests are the most widely used diagnostic tests in the workup of patients with viral pneumonia [6].

Over the last several decades, there has been an increase in the knowledge base and general awareness of viral pneumonia due to multiple epidemics and pandemics involving lower respiratory tract infections of viral origin. Additionally, there is a growing population at increased risk of viral pneumonia, increased prevalence of immunosuppressive states, and an increased realization of bacterial and viral co-infection necessitating a higher clinical index of suspicion and early identification of respiratory viruses [7]. The effect of these various factors on pneumonia and other diseases has been clearly described [8,9].

Variations in the distribution and outcomes of viral pneumonia based on race and ethnic demographics have been demonstrated in the United Kingdom, Australia, and New Zealand [10-12]. A review of existing literature reveals a relative paucity of studies investigating racial distribution and variations in hospitalizations and deaths resulting from viral pneumonia in the United States. To bridge this knowledge gap, we used the two most recent releases of the Nationwide Inpatient Sample (NIS) to compare outcomes of patients with viral pneumonia by race with primary emphasis on inpatient mortality.

## Materials and methods

Design and data source

This was a retrospective cohort study in the US between January 1, 2016 and December 31, 2017. The NIS, the largest database of hospital inpatient stays in the United States derived from billing data submitted by hospitals to statewide data organizations across the US, was used [13,14]. It approximates a 20% stratified sample of discharges from US community hospitals, which is weighted to obtain national estimates [15]. The International Classification of Diseases, Tenth Revision, Clinical Modification/Procedure Coding System (ICD-10-CM/PCS) was used in the coding. Diagnoses are divided into a principal diagnosis; the main ICD-10 code for the hospitalization, and secondary diagnoses which are discharge diagnoses other than the principal diagnosis.

Study population

We queried the NIS 2016 and 2017 database for patients 18 years and above who had a principal discharge diagnosis of viral pneumonia including influenza due to identified novel influenza A virus with pneumonia (J09.X1), influenza due to other identified influenza virus with pneumonia (J10.0), influenza due to unidentified influenza virus with pneumonia (J11.0), and viral pneumonia, not elsewhere classified (J12). Patients were excluded if they had viral pneumonia only as a secondary diagnosis. The NIS provides data on racial distribution of the hospitalizations: Race, uniform coding: (1) White, (2) Black, (3) Hispanic, (4) Asian or Pacific Islander, (5) Native American, (6) other. We combined groups 4, 5, and 6 into “others”, forming a modified racial grouping as employed in prior NIS based publications [16-21]. 

Outcome measures

The primary outcome was comparing inpatient mortality among patients principally admitted for viral pneumonia by race. Secondary outcomes in this population included the odds of having a secondary discharge diagnosis of sepsis, septic shock, acute respiratory failure, acute respiratory distress syndrome, non-ST segment elevation myocardial infarction (NSTEMI), acute kidney failure, deep vein thrombosis, pulmonary embolism, cerebrovascular accident, need for mechanical ventilation, vasopressors as well as mean length of hospitalization and mean total hospital charges.

Statistical analysis

Stata® Version 16 software (StataCorp., College Station, TX, USA) was used for analysis. All analyses were conducted using the weighting samples for national estimates in adjunct with Healthcare Cost and Utilization Project regulations for using the NIS database. Co-morbidities were calculated as proportions of the cohort and the Chi-squared test was used to compare these characteristics between the racial subgroups. A univariate screen was done to confirm whether confounders affected outcomes, with variables having a p-value less than 0.2 included in the multivariate regression analysis. Multivariate regression analysis was then done to adjust for these confounders while calculating the primary and secondary outcomes. The validated MuLBSTA Score for viral pneumonia as well as the Pneumonia Severity Index was used to identify confounders [22,23]. A p of <0.05 was set as the level for statistical significance.

Ethical considerations

The NIS is a retrospective database lacking individual or hospital identifiers. This study was therefore exempt from our Institutional Review Board approval.

## Results

Patient characteristics

The combined NIS database for 2016 and 2017 contained over 71 million weighted hospital discharges of which 89,650 satisfied the inclusion criteria for the study. These patients were adults with a principal discharge diagnosis of viral pneumonia.

White patients accounted for over two-thirds (68.2%) and they were significantly older (70.1 vs 58.4 and 64.2 years, p<0.001) compared to Blacks and Hispanics. The patients were predominantly females and were majorly insured through Medicaid. Hispanics had the highest proportion of diabetics (44.5% vs 27.9% and 39.5%), Blacks had the highest proportion of obesity, chronic disease, and comorbid current or history of malignancy, while Whites had the most proportion of hypertension, smoking history, and history of chronic obstructive pulmonary disease. Patient and hospital characteristics are detailed in Table 1.

**Table 1:** Distribution of patient and hospital characteristics of viral pneumonia hospitalizations by race #: for 2017. *: Co-morbidities were secondary diagnoses. CHF: Congestive heart failure, CKD: Chronic kidney disease, COPD: Chronic obstructive pulmonary disease, CVA: Cerebrovascular accident, IHD: Ischemic heart disease

Variable	Whites (%)	Blacks (%)	Hispanics (%)	Others (%)	p-value
	n= 61,110 (68.2)	n= 10,225 (11.4)	n= 8,855 (9.9)	n= 9,460 (10.5)	
Patient characteristics					
Age (in years), mean	70.1	58.4	64.2	67.7	<0.001
Women	53.9	60.7	56.4	54.9	<0.001
Insurance type					
Medicaid	71.4	52.5	56.8	62.4	
Medicare	63.4	20.1	18.9	14.0	
Private	20.2	21.0	18.3	20.4	
Uninsured	2.0	6.4	6.0	3.2	
Charlson Comorbidity Index score					<0.001
0	19.3	18.7	18.3	18.7	
1	27.9	23.4	25.9	27.2	
2	19.6	17.0	19.0	19.6	
≥3	33.2	40.9	36.8	34.5	
Median annual income in patient’s zip code, US$^#^					<0.001
1-43,999	23.2	54.4	41.5	23.2	
44,000-55,999	26.8	19.6	25.1	23.5	
56,000-73,999	26.0	16.1	20.1	28.4	
≥74,000	24.0	9.9	13.3	24.9	
Co-morbidities*					
Diabetes	27.9	39.5	44.5	34.4	<0.001
Hypertension	42.3	39.7	39.3	38.9	0.002
Smoking history	39.8	37.8	30.7	32.6	<0.001
CHF	22.9	23.4	22.3	22.0	0.706
CKD	15.2	24.6	18.4	17.9	<0.001
Obesity	16.1	23.9	19.3	14.0	<0.001
Chronic IHD	25.5	18.4	21.6	21.1	<0.001
Prior CVA	2.0	3.0	2.7	2.1	0.016
COPD	28.4	20.4	18.5	20.9	<0.001
Dependent on O_2_	7.3	5.6	4.9	5.7	<0.001
Liver disease	2.8	2.6	4.8	4.0	<0.001
Anemia	22.3	32.4	26.3	26.5	<0.001
Malignancy	37.1	44.1	38.5	40.7	<0.001
Hospital characteristics					
Hospital region					<0.001
Northeast	19.4	19.0	16.5	15.5	
Midwest	28.0	22.5	8.5	23.7	
South	33.7	51.1	42.3	21.6	
West	18.9	7.4	32.7	39.2	
Hospital bed size					0.001
Small	23.8	19.3	21.9	20.9	
Medium	28.1	26.1	29.6	25.8	
Large	48.1	54.6	48.5	53.3	
Urban location	85.9	94.1	94.8	85.9	<0.001
Teaching hospital	60.0	75.7	69.2	62.3	<0.001

Primary outcome: in-hospital mortality

The in-hospital mortality for viral pneumonia was 2.22% of the total cohort. The crude mortality rate was highest among Whites (2.55%). Both Blacks and Hispanics had lower adjusted odds ratios of inpatient mortality (aOR: 0.39, 95% CI 0.229 - 0.662, p<0.001 and aOR: 0.55, 95% CI 0.347 - 0.858, p=0.009, respectively) compared to Whites. We made adjustments for both hospital and patient variables including comorbidities.

Secondary outcomes

The total length of hospitalization and the total hospital charges between the Blacks and Hispanic groups were compared to Whites using multivariate linear regression model. Blacks and Hispanics had mean decreases in length of hospitalization (-0.5, CI: (-0.761 - [-0.196], p=0.001 and (-0.3, CI: (-0.597 - [-0.103], p=0.006, respectively) compared to Whites. There was however no difference in mean total hospital charge between them. Black and Hispanic patients were found to have lower adjusted odds ratio of having acute respiratory failure (aOR: 0.54, 95% CI: 0.471 - 0.614, p<0.001, and 0.66, 95% CI: 0.576 - 0.753, p<0.001 respectively). However, Blacks had higher odds of developing acute kidney failure (aOR: 1.20, 95% CI: 1.043 - 1.386, p=0.011) when compared to Whites. Detailed outcomes are provided in Tables 2, 3.

**Table 2:** Clinical outcomes of hospitalizations for viral pneumonia by race ARDS: Acute respiratory distress syndrome, NSTEMI; Non-ST segment elevation myocardial infarction

Outcome	Whites (%)	Blacks (%)	Hispanics (%)	Others (%)
Primary outcome				
In-hospital mortality	(2.6)	(1.0)	(1.4)	(2.2)
Secondary outcomes				
Length of stay, mean	5.2	5.2	5.0	5.2
Total hospital charges, mean US$	43378	49697	54922	55769
Sepsis	2.9	2.5	3.6	4.9
Septic shock	0.9	1.0	1.0	1.4
NSTEMI	1.9	1.4	1.9	2.0
Mechanically ventilated	3.7	4.4	4.0	4.0
Used pressors	1.8	2.6	2.5	4.7
Acute kidney failure	17.1	20.9	17.3	17.8
Acute respiratory failure	27.2	17.8	21.0	26.7
ARDS	0.8	0.5	0.7	1.1
Deep vein thrombosis	1.2	1.7	0.9	1.3
Pulmonary embolism	0.5	0.7	0.5	0.3
Cerebrovascular accident	0.3	0.4	0.4	0.4

**Table 3:** Adjusted odds ratio of clinical outcomes of Blacks and Hispanics with viral pneumonia compared to Whites *; statistically significant, #; adjusted mean difference, aOR: adjusted odds ratio, CI: confidence interval, ARDS: Acute respiratory distress syndrome, NSTEMI; Non-ST segment elevation myocardial infarction

Outcome	Blacks		Hispanics	
	aOR (95% CI)	p-value*	aOR (95% CI)	p-value*
Primary outcome				
In-hospital mortality	0.39 (0.229 – 0.662)	<0.001*	0.55 (0.347 – 0.858)	0.009*
Secondary outcomes				
Length of stay, mean	-0.5^#^ (-0.761 – [-0.196])	0.001*	-0.3^#^ (-0.597 – [-0.103])	0.006*
Total hospital charges, mean US$	-1736^#^ (-6452 - 2981)	0.471	4377^#^ (-542 - 9297)	0.081
Sepsis	0.60 (0.423 – 0.837)	0.003*	1.04 (0.783 – 1.382)	0.784
Septic shock	0.68 (0.377 – 1.224)	0.198	0.80 (0.453 – 1.403)	0.433
NSTEMI	0.82 (0.523 – 1.288)	0.389	1.07 (0.723 – 1.595)	0.724
Mechanically ventilated	0.81 (0.625 – 1.061)	0.128	0.92 (0.699 – 1.205)	0.536
Used pressors	1.07 (0.739 – 1.554)	0.715	1.39 (0.932 – 2.069)	0.106
Acute kidney failure	1.20 (1.043 – 1.386)	0.011*	0.94 (0.811 – 1.099)	0.458
Acute respiratory failure	0.54 (0.471 – 0.614)	<0.001*	0.66 (0.576 – 0.753)	<0.001*
ARDS	0.45 (0.230 – 0.866)	0.017*	0.52 (0.273 – 1.009)	0.053
Deep vein thrombosis	1.35 (0.870 – 2.030)	0.183	0.68 (0.385 – 1.202)	0.184
Pulmonary embolism	1.14 (0.598 – 2.202)	0.679	0.92 (0.435 – 1.961)	0.835
Cerebrovascular accident	1.52 (0.600 – 3.861)	0.378	1.18 (0.479 – 2.983)	0.714

## Discussion

The crude mortality rate of the total population for viral pneumonia according to our study was 2.22%. This rate was highest among Whites (2.55%), while Blacks and Hispanics comparatively had statistically significant lower odds of inpatient mortality after adjustment for possible confounders. We also found that Blacks and Hispanics had significantly shorter lengths of hospital stay and decreased odds of acute respiratory failure, a known complication of viral pneumonia [24]. There was however no significant difference in total hospital charges accrued between the three cohorts.

The rather surprising findings of our study are contradicted by the results of a study conducted by Hausmann et al. which revealed that mortality rate associated with CAP for patients at hospitals with the majority racial composition of those attended by average African Americans (OR = 1.21; 95% CI = 1.18 - 1.25) or Hispanics (OR = 1.18; 95% CI = 1.14 - 1.23) was higher than for patients at hospitals with the majority racial composition of those attended by average Whites [25]. A systematic review conducted by Pan et al. in the UK buttressed this variance and showed that Blacks, Asians, and other minority ethnic groups were at increased risk of acquiring severe acute respiratory syndrome coronavirus 2 (SARS-CoV-2) and poorer clinical outcomes from the disease [26]. However, these studies analyzed all patients with pneumonia even though there are known differences in management and outcomes of bacterial pneumonia as against viral pneumonia. The prognosis of viral pneumonia has been shown to be worse in older adults, those with a history of smoking, and patients with chronic obstructive pulmonary disease (COPD) [26,27]. These factors were more prevalent among Whites in our study group. To further elucidate possible explanations for these outcomes, we recommend more large-scale studies into racial distributions and variations in outcomes of patients with viral pneumonia.

Our study has several strengths. First, the data was sourced from a large nationwide dataset, to provide a large sample size that enabled us to compare mortality outcomes despite the low inpatient mortality rate associated with this cohort of patients. Second, the nature of the database allows us to provide insights into the comparison of baseline demographics and hospital outcomes between different racial groups to statistically significant levels.

There are some limitations to the study. NIS database studies are subject to non-randomization. NIS database deals with hospitalizations, not individual patients, so patients admitted multiple times will be counted multiple times [28]. There was no reliable way to determine if the secondary diagnoses preceded or developed during the index hospitalization [29]. Additionally, laboratory and radiologic data that could indicate underlying disease severity and inflammatory activity are not available in the NIS database [30].

## Conclusions

In summary, among adults, Blacks and Hispanics are at lower risk of adverse outcomes including length of hospital stay, respiratory complications, and inpatient mortality when compared to Whites with viral pneumonia. This racial discrepancy identified warrants further randomized studies into possible unadjusted confounders to address healthcare disparities.
